# Age-by-disease biological interactions: implications for late-life depression

**DOI:** 10.3389/fgene.2012.00237

**Published:** 2012-11-16

**Authors:** Brandon C. McKinney, Hyunjung Oh, Etienne Sibille

**Affiliations:** ^1^Department of Psychiatry, University of PittsburghPittsburgh, PA, USA; ^2^Center for Neuroscience, University of PittsburghPittsburgh, PA, USA

**Keywords:** late-life depression, depression, molecular aging, gene expression, telomere, oxidative stress, epigenetic modifications

## Abstract

Onset of depressive symptoms after the age of 65, or late-life depression (LLD), is common and poses a significant burden on affected individuals, caretakers, and society. Evidence suggests a unique biological basis for LLD, but current hypotheses do not account for its pathophysiological complexity. Here we propose a novel etiological framework for LLD, the age-by-disease biological interaction hypothesis, based on the observations that the subset of genes that undergoes lifelong progressive changes in expression is restricted to a specific set of biological processes, and that a disproportionate number of these age-dependent genes have been previously and similarly implicated in neurodegenerative and neuropsychiatric disorders, including depression. The age-by-disease biological interaction hypothesis posits that age-dependent biological processes (i) are “pushed” in LLD-promoting directions by changes in gene expression naturally occurring during brain aging, which (ii) directly contribute to pathophysiological mechanisms of LLD, and (iii) that individual variability in rates of age-dependent changes determines risk or resiliency to develop age-related disorders, including LLD. We review observations supporting this hypothesis, including consistent and specific age-dependent changes in brain gene expression and their overlap with neuropsychiatric and neurodegenerative disease pathways. We then review preliminary reports supporting the genetic component of this hypothesis. Other potential biological mediators of age-dependent gene changes are proposed. We speculate that studies examining the relative contribution of these mechanisms to age-dependent changes and related disease mechanisms will not only provide critical information on the biology of normal aging of the human brain, but will inform our understanding of age-dependent diseases, in time fostering the development of new interventions for prevention and treatment of age-dependent diseases, including LLD.

## INTRODUCTION

Among elderly individuals, depressive symptoms are common and burdensome. Approximately 1% of individuals over the age of 65 meet criteria for major depressive disorder (MDD), as defined by the diagnostic and statistical manual of mental disorders, fourth edition, text revision (DSMIV-TR; American Psychiatric Association, 2000), a prevalence lower than that in younger adults (Kessler et al., 2003). Another 15–25%, however, experience depressive symptoms that, while not meeting criteria for MDD, do cause significant distress and interfere with daily functioning (Koenig and Blazer, 1992). In this article, the term late-life depression (LLD) will be used to refer to individuals over the age of 65 who for the first time in their lives meet criteria for MDD or display clinically significant depressive symptoms. Individuals with LLD experience greater functional disability (Dombrovski et al., 2007) and cognitive decline (Lenze et al., 2005) than those without. Further, they are at increased risk of morbidity and mortality from medical illness (Ganguli et al., 2002). LLD also appears to contribute to increased rates of suicide among older individuals (Van Orden and Conwell, 2011).

The biological substrates of LLD are being characterized and several hypotheses for the etiology and pathophysiology of LLD have been proposed, including the vascular hypothesis (Alexopoulos et al., 1997), inflammation hypothesis (Alexopoulos and Morimoto, 2011), and dementia prodrome hypothesis (Byers and Yaffe, 2011; reviewed in McKinney and Sibille, 2012). Here, we propose an alternative and complementary hypothesis, which we termed the age-by-disease biological interaction hypothesis of LLD. Central to this hypothesis is the concept of molecular aging of the human brain. An earlier version of this hypothesis has been described elsewhere (McKinney and Sibille, 2012).

## MOLECULAR AGING OF THE HUMAN BRAIN

Despite its critical importance to a population that is growing older, “normal” brain aging is understudied. This may be due to the often expressed, but false belief held by many that aging is inescapable, broad-ranging and non-specific. Studies that have investigated biological aging have revealed specific changes and thus avenues for intervention. At the cellular level in the human brain, morphological and stereological studies reveal a decrease in neuron volumes, a small loss or no change in cell numbers (Morrison and Hof, 1997; Pakkenberg and Gundersen, 1997), and a progressive thinning of cortical thickness, affecting both gray and white matter (Resnick et al., 2003; Sowell et al., 2003). Similar structural changes with age have been demonstrated in the brains of animal models (Jucker and Ingram, 1997; Peters, 2002). At the molecular level in animal models, less than 10% of brain-expressed genes exhibit age-related changes in gene expression (Lee et al., 1999, 2000; Jiang et al., 2001; Blalock et al., 2003; Sibille et al., 2007). Similar numbers have been reported in studies of human tissue (Lu et al., 2004; Avramopoulos et al., 2011). In one such study of human tissue from the prefrontal cortices of subjects aged 13–79, our group used gene microarray technology to investigate age-related changes in gene expression and reported that approximately 7.5% of genes changed significantly with aging (Erraji-Benchekroun et al., 2005). Other studies have confirmed these results, identifying a maximum of ~10% of all detected genes, depending on sample size and analytical power of the respective studies (Yankner et al., 2008; Glorioso et al., 2011). Of note, not only is the identity of the genes and gene classes that are affected with aging consistent among studies, but so are the directions of change.

Interestingly, the identity of the genes whose expression changes with age suggests that specific cellular populations and biological processes are selectively affected by the aging process. For instance, expression of genes playing a role in glial-mediated inflammation, oxidative stress responses, mitochondrial function, synaptic function and plasticity, and calcium regulation and neuropeptide signaling have consistently been shown across multiple studies to be affected by aging, while numerous other neuronal and glial genes remain apparently unchanged (Yankner et al., 2008; Glorioso and Sibille, 2011). Overall, age-upregulated genes are mostly of glial origin and related to inflammation and cellular defenses, while downregulated genes display mostly neuron-enriched transcripts relating to cellular communication and signaling (Erraji-Benchekroun et al., 2005).

Using the expression levels of the age-dependent genes and their expected trajectories with age, we have generated predicted ages of individual subjects from which the brain tissue was sampled, and demonstrated that this predicted age is highly correlated with the chronological age of that individual (Erraji-Benchekroun et al., 2005; Glorioso and Sibille, 2011; Glorioso et al., 2011). We have termed this predicted age the “molecular age”. These findings suggest that gene expression changes with age can be used as biomarkers for brain aging.

## MOLECULAR AGING AND BRAIN-RELATED DISEASE PATHWAYS

**FIGURE 1:**
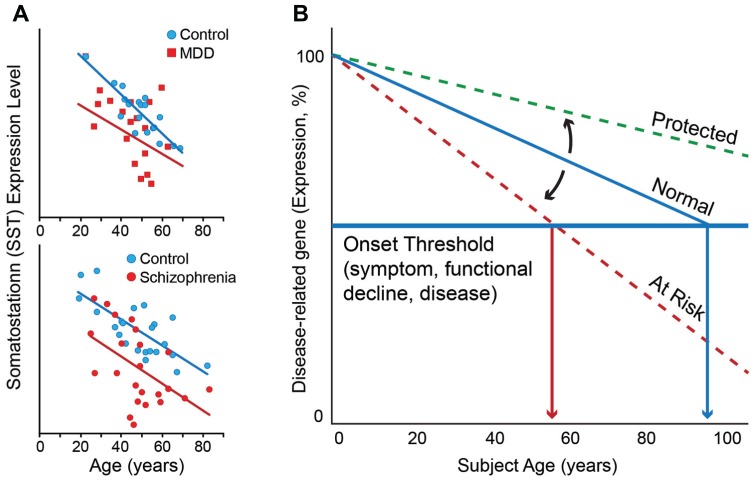
**(A)** Somatostatin expression decreases with age in control subjects. Individuals with MDD (top panel) and schizophrenia (bottom panel) display lower levels of expression than control subjects at most ages, together prompting the hypothesis that decreased expression of somatostatin in depression and schizophrenia may represent early brain age-related molecular phenotypes in these individuals, which would render these subjects more vulnerable to developing psychiatric diseases. **(B)** Based on similar age and disease observations for numerous additional genes, we have proposed a model of “age-by-disease biological interaction” (Glorioso et al., 2011). In this model, change in expression of disease-related genes (a decrease is shown) across a threshold (horizontal blue line) marks the onset of disease symptoms. Changes in the trajectory of age-related changes in expression of disease-related genes (Y-axis) determine at what age (X-axis), or even if, an individual develops disease symptoms (vertical red arrows). Modulators (black arrows) may thus place an individual on “at risk” or “protected” trajectory. Per this model, individuals with LLD may have modulators, genetic or environmental, that place them on an “at risk” trajectory for developing mood symptoms. Figures are adapted from Morris et al. (2008), Tripp et al. (2011), Glorioso and Sibille (2011), and McKinney and Sibille (2012).

This correlation between molecular and chronological ages is robust in individuals without neurodegenerative and neuropsychiatric disorders (Erraji-Benchekroun et al., 2005), but investigations of individual genes suggest that the molecular age or individual gene trajectory can deviate from chronological age in individuals with these disorders. To illustrate this phenomenon, one can look at somatostatin (SST), a signaling neuropeptide that is expressed in a subpopulation of gamma-aminobutyric acid (GABA)-positive inhibitory interneurons (Viollet et al., 2008). We have demonstrated that expression of SST decreases progressively with age in individuals without neurodegenerative and neuropsychiatric disorders such that expression levels at 70 years of age are approximately 40–50% of those at 20 years of age (Erraji-Benchekroun et al., 2005; Tripp et al., 2011; **Figure 1A**). SST is also downregulated in individuals with MDD. Interestingly, the magnitude and direction of change in SST expression with age is similar to that of control individuals, but the absolute values of SST expression are lower at most ages in individuals with MDD compared to those without (Sibille et al., 2011; Tripp et al., 2011; **Figure 1A**, top panel). Similar findings have been observed in subjects with schizophrenia (Morris et al., 2008; **Figure 1A**, bottom panel). 

The relationship between gene expression of a disorder-associated gene and aging is not limited to SST. In fact, genome-wide investigations have reported that up to a third of age-regulated genes in the human brain have been at some point associated in the literature with neurodegenerative (Alzheimer’s, Parkinson’s, and Huntington’s diseases, amyotrophic lateral sclerosis) and neuropsychiatric disorders (bipolar depression, major depression, and schizophrenia; see Figure 3 in Glorioso et al., 2011 for details). Not only do the genes relevant to these brain disorders show age-related changes, but the direction of the changes that occur with age is almost always in the direction thought to cause or promote diseases. See Table 1 in Glorioso et al. (2011) for a list of approximately 40 top candidate disease genes that exhibit agreement between age- and disease-related changes. Studies of gene expression in Alzheimer’s disease (AD) and normal aging find that most of the changes in gene expression that occur in normal aging also occur in AD, only in greater magnitude (Miller et al., 2008; Avramopoulos et al., 2011). The difference in the magnitude of change in gene expression between individuals with brain disease and those without during aging is attributable to the disease. Our hypothesis posits that it is these changes that drive the disease process; however, it cannot be ruled out that these additional changes in magnitude are the result of the disease process rather than the cause. That said, there are certainly genes for which it is known that the age-related direction of change in gene expression directly causes, rather than is a consequence of, the disease, for example, the Parkinson’s disease genes PINK_1_ and DJ_1_/PARK_7_. The expression of these genes progressively declines with age and it is known that individuals with loss of expression/function mutations in these genes develop Parkinson’s disease (Schapira, 2008). In contrast, relatively fewer of the larger pool of genes that do not display age-dependent changes (<5%), are associated with neurodegenerative and neuropsychiatric diseases.

Recently, we have further investigated the relationship between age, gene changes, and neuropsychiatric disorders, specifically in the context of MDD (Douillard-Guilloux et al., 2012). Results demonstrated that most MDD-related genes were frequently age-regulated in both MDD and in control subjects, and that the effects of MDD and age were positively correlated. Moreover, most genes that are age-dependent in control subjects displayed greater age effects in MDD subjects, and the overall increased prevalence of age effects on genes in MDD subjects corresponded to similar trends in controls, rather than representing *de novo* age effects. This systematic correlation between age-dependent and depression-related changes, with greater effects in depressed subjects, further suggests that normal brain aging is a risk factor for biological changes observed in MDD subjects.

One interpretation of these observations is that age-dependent changes (i.e., molecular aging) are on an earlier trajectory in individuals who develop MDD and potentially other neuropsychiatric disorders. However, it is important to note that these studies are cross-sectional and do not follow the longitudinal progression of gene changes within individuals, so it is not known whether age-dependent changes are on an earlier trajectory, or whether changes occurred at earlier time points and were fixed at lower levels, for instance in the case of SST. So while we hypothesize that age may be pushing the expression of genes in disorder-causing directions, an alternate scenario is that of earlier and fixed changes, which then act as latent vulnerability factors that are revealed with advancing age, resulting in increased vulnerability to develop neurodegenerative and psychiatric disorders, including LLD.

## AGE-RELATED CHANGES IN GENE EXPRESSION APPEAR TO BE, IN PART, GENETICALLY MODULATED

While molecular and chronological ages are highly correlated, we have also reported individual deviations from predicted ages (Erraji-Benchekroun et al., 2005; Glorioso et al., 2011). The fact that molecular age can deviate from its chronological age suggests that modulating factors exist and may contribute to one’s vulnerability to brain aging and to developing late-life brain disorders, such as LLD. In the age-by-disease biological interaction hypothesis we have proposed that those individuals with older predicted molecular ages compared to their chronological age may not only display greater biological brain aging, but may also be at greater risk of age-gated brain diseases, because gene expression of disease-related genes would have proceeded further in disease-promoting directions. Conversely, subjects with younger age-dependent gene trajectories and lower predicted molecular ages would be at lower risk, and may in fact display resiliency against LLD and other late-life disorders (**Figure 1B**). Environmental and genetic factors represent obvious candidates to modulate the trajectory of biological aging.

In a proof-of-principle study, our laboratory sought to demonstrate a genetic role in modulating the aging process. The above-described “molecular age” assay was used to characterize the brain tissue of individuals carrying different polymorphisms of the sirtuin genes (Glorioso et al., 2011), a family of genes previously demonstrated to modulate age and longevity in nematodes, insects, and rodents. We focused on SIRT5, due to prior report of altered expression for that gene in a rodent model of anticipated brain aging (Sibille et al., 2007). This study found that subjects carrying a low-expressing polymorphism of the SIRT5 gene had molecular ages that were older than actual chronological age, as measured in the three different areas of the cerebral cortex (i.e., BA9, BA24, and BA47) of human postmortem samples (Glorioso et al., 2011). Interestingly, this effect was not demonstrated in the amygdala, a brain area in which the low-expressing polymorphism of the SIRT5 gene does not appear to affect expression levels of SIRT5 as it does in the cerebral cortex. These findings at the molecular level are consistent with findings from studies of brain structure with age that show robust decreases in gray matter in the cerebral cortex but more variable decreases in the amygdala (Good et al., 2001). The effect of the low-expressing SIRT5 polymorphism on molecular age was accompanied by expression changes for a set of genes whose products are localized to the mitochondria, including PINK-1 and DJ-1, two Parkinson’s disease-associated genes, in ways that would promote mitochondrial dysfunction-related diseases, including Parkinson’s disease. This (correlative) proof-of-principle study suggests that factors that affect brain aging can potentially place an individual at higher risk for disease, through a mechanism by which it accelerates brain biological aging, and thus promotes changes in expression of disorder-relevant genes in disease-causing directions. With respect to **Figure 1B**, the low-expressing polymorphism of the SIRT5 gene can be thought of a modulator that puts one on the “at risk” trajectory. The converse of this model is that factors delaying age trajectories of gene changes may lead to younger brain molecular aging and potential resiliency toward developing functional declines and age-related disorders, including LLD (**Figure 1B**).****

## PUTATIVE MECHANISMS FOR AGE-RELATED CHANGES IN GENE EXPRESSION

The mechanisms by which age-related changes in gene expression occur are unknown. Candidate mediators include among others, loss of telomere integrity, increased oxidative stress, and epigenetic modifications.

### LOSS OF TELOMERE INTEGRITY

Telomeres are regions of repetitive nucleotide sequences at each end of a chromosome. One of the hypothesized functions of telomeres is to deter the degradation of genes near the ends of chromosomes by instead allowing repetitive telomeres to shorten, a necessary part of chromosome replication. Telomeres are highly susceptible to oxidative stress because of their high content of guanines. As both chromosome replication and oxidative stress increase with age, one would expect telomeres to shorten with increased age. Indeed, in peripheral tissues, it has been consistently demonstrated that telomeres become shorter as one ages and once telomeres reach a critical length, irreversible arrest of cell division or apoptosis is triggered (Hayflick and Moorhead, 1961; Chin et al., 1999; Sharpless and DePinho, 2004; Flores and Blasco, 2009; Sahin and DePinho, 2012). Although telomere shortening has not yet been observed in the human brain, studies suggest that peripheral telomere length is a biomarker for aging. Leukocyte telomere shortening is positively correlated with the chronicity of stress and depression (Epel et al., 2004; Wolkowitz et al., 2011a) and is associated with incidence of age-related diseases such as myocardial infarction, stroke, and shorter lifespan (reviewed in Wolkowitz et al., 2011b). It is not known whether telomere shortening increases the risk of LLD, however, given the common pathway of aging and depression, the possibility cannot be excluded.

Recent animal studies suggest that the putative link between telomere integrity and depression-like behaviors extends to the brain, and that this link may be mediated by telomerase activity (Zhou et al., 2011). In that study, the expression of telomerase was decreased in the hippocampi of mice subjected to chronic mild stress, and hippocampal infusion of a telomerase inhibitor induced depressive-like behaviors that did not respond to antidepressant treatment (Zhou et al., 2011). Given that neurogenesis has been implicated in antidepressant responses in mice (Santarelli et al., 2003) and that the proliferation capacity of neural stem cells highly depend on telomerase activity (Ferron et al., 2009), the authors suggested telomerase activity may play a role in linking mechanisms of aging and depression (Zhou et al., 2011); although the translation of these observations to humans is contentious, due to very low rates of neurogenesis in adult human subjects (Bhardwaj et al., 2006).

The anti-apoptotic role of telomerase is thought to reflect its capacity of maintaining DNA integrity, however, recent studies have reported other putative functions (reviewed in Saretzki, 2009). Overexpression of telomerase reverse transcriptase (TERT), protects mouse neurons from excitotoxicity by improving basal mitochondrial membrane potential and Ca^2^^+^ uptake into mitochondria (Kang et al., 2004) and decreases cellular reactive oxygen species (ROS; Ahmed et al., 2008). Furthermore, TERT mediates the neurotrophic action of BDNF (Fu et al., 2002) and affects the global pattern of gene expression in the direction of cell survival, including upregulation of growth promoting genes (FGF5, EGFR, and etc.) and downregulation of cell-growth inhibitors such as IGFBPs (Smith et al., 2003; Choi et al., 2008). Changes in telomerase activity with age and MDD have yet to be explored in the human brain. However, given the fact that neurotrophic growth signaling, including reduced BDNF, is decreased in MDD (Sen et al., 2008; Guilloux et al., 2011; Tripp et al., 2011), we cannot exclude the possibility that telomeres and telomerase activity may significantly contribute to the mediation of stress and brain aging.

### INCREASED OXIDATIVE STRESS

Oxidative stress is the damage caused to cells as a result of an imbalance between the production of ROS and the ability of the cells to reduce the ROS or repair the resulting damage. The degree of oxidative stress to cellular components, including DNA, correlates positively with age (Joseph et al., 2005; Epel, 2009; Wolkowitz et al., 2011b). Because of their high demand for energy and postmitotic status, neurons appear to be particularly sensitive to oxidative stress and thus aging.

One way in which oxidative stress may contribute to age-related changes in gene expression is via its direct effect on DNA. Lu et al. (2004) showed that age-related decrease in gene expression is related to the accumulation of oxidative DNA damage. The underlying mechanism was suggested that promoter regions with high GC contents are specifically vulnerable to oxidative damage. Oxidated promoter regions may potentially adopt different conformation and lose affinity for transcription factors (Lu et al., 2004). Damage on mitochondrial DNA (mtDNA), which is considered extremely vulnerable to oxidation due to its proximity to the site of ROS production, respiratory chain, and the absence of protective histone (Lee and Wei, 2007), results in downregulation of genes related to respiratory chain and further, energy metabolism impairment (Lin et al., 2002). Several studies showed that psychiatric diseases including MDD, bipolar disorder, and schizophrenia are associated with mitochondrial dysfunction (Rezin et al., 2009) and that accumulation of mtDNA damage induces mood disorder-like phenotypes as well as premature aging in mice (Trifunovic et al., 2004; Kasahara et al., 2006). These results support the idea that oxidative stress plays a role as a link between aging and depression.

Another direct way in which oxidative stress may contribute to age-related changes in gene expression is via its effect on transcription factors. For example, ROS are able to activate nuclear factor kappa B (NF-κB) by decreasing binding affinity of the inhibitory subunit, Iκ-B, to NF-κB, an observation made relevant by the fact that NF-κB activity has been demonstrated to increase with aging and depression (Toliver-Kinsky et al., 1997; Koo et al., 2010). Also, transcription factors containing the zinc-finger DNA binding motif appear to be especially susceptible to damage from oxidative stress due to their high cysteine residue content. As intracellular ROS accumulate, oxidation of the thiol residues in cysteine occurs and binding affinity for DNA is lost. One of the zinc-finger transcription factors, Sp1, an ubiquitous transcription factor for housekeeping genes and enzymes involved in glucose metabolism and DNA synthesis, has been demonstrated to have decreased DNA binding affinity with advancing age (Ammendola et al., 1992, 1994; Wu et al., 1996). Interestingly, genes with lower affinity binding sites to Sp1 are more influenced by oxidative stress than those containing high-affinity sites (Ammendola et al., 1992, 1994; Wu et al., 1996). Furthermore, ROS decreases telomerase activity. Antioxidant treatment normalizes catalytic activity of TERT and delays cellular senescence (Haendeler et al., 2004).

In addition to the conformational change of gene and transcription factors, ROS can act on various cellular signaling pathways to control gene expression. For example, ROS increase p53 signaling, which has been implicated in various neurodegenerative diseases and thought to mediate its effect by increasing expression of genes related to cell cycle arrest, DNA repair, and apoptosis in response to cellular stressors such as DNA damage and hypoxia (Lundberg et al., 2000). Another example is illustrated by p38 MAP Kinase (MAPK). When p38 MAPK is activated by oxidative stress, it promotes lamin B1 accumulation and expression of several transcription factors related to cellular senescence and apoptosis such as p53, CREB, C/EBPβ, and ATF2 (Wagner and Nebreda, 2009; Barascu et al., 2012). Interestingly, selective p38 MAPK deletion in serotonergic neurons produces stress resilience in an animal model of depression by inhibiting serotonin transporter translocation to plasma membrane (Bruchas et al., 2011).

In summary, the main effect of oxidative stress on aging has been thought to be the accumulation of toxic, inactive molecules produced randomly by ROS. However, oxidative stress may also have an active role in aging and related diseases, through direct modification of DNA and transcription factor integrity and through indirect pathways regulating upstream modulators. These observations suggest that antioxidants may contribute to preventing biological changes and/or associated symptoms of depression, in addition to their potential anti-aging effects.

### EPIGENETIC MODIFICATIONS

Epigenetic modifications, including DNA methylation and histone modification, regulate gene expression without changing the primary DNA sequence. Though classically viewed as a permanent event, recent data indicates that such modifications are influenced by genetic and environmental factors in adult organisms, including changes in methylation patterns across the lifespan (Numata et al., 2012). At the genome level, DNA methylation decreases with age. In contrast, CpG islands of many specific promoter regions that are typically not methylated become methylated with aging, including in promoters of tumor suppressor genes, estrogen receptor (ER), and insulin-like growth factor 2 (IGF2; Issa et al., 1994, 1996; So et al., 2006; Numata et al., 2012). Changes in DNA methylation at the glucocorticoid receptor, potentially due to early-life stress, was also correlated with altered vulnerability to psychiatric disorders and death by suicide in adults (McGowan et al., 2009).

Similarly, histone modifications such as acetylation, phosphorylation, symoylation, and methylation change with age. It was recently demonstrated that the aging-related deficit of long-term synaptic plasticity in the rodent hippocampus is related to decreased BDNF expression secondary to decreased acetylation of histones residing at the BDNF promoter region (Zeng et al., 2011). This observation fits well with human studies demonstrating reduced BDNF function in aging and depression (Webster et al., 2002; Guilloux et al., 2011). Also, changes in histone modifications in rodent systems may be protective against the effects of stress (Covington et al., 2011; Uchida et al., 2011). In support of a specific role for histone acetylation in age-related changes in gene expression, sirtuins (NAD-dependent histone deacetylases) have been implicated in longevity in yeast and invertebrates (Longo and Kennedy, 2006). A recent rodent study directly links SIRT1 to the risk of anxiety-like behaviors through its activity on monoamine oxidase A and serotonin levels (Libert et al., 2011). In the same study, the authors reported association between a single nucleotide polymorphism in the human SIRT1 gene and the risk of various psychiatric disorders such as anxiety disorder, panic disorder, and major depression. Furthermore, overexpression of SIRT6 in mice has been reported to increase lifespan and protect from diet-induced physiological damage (Kanfi et al., 2010, 2012) and SIRT6 knockout mice exhibit accelerated aging. Interestingly, decreased expression of SIRT6 has been observed during MDD (Abe et al., 2011). How SIRT6 mediates its effect on aging or is involved in MDD is not clear, but its functions as an HDAC (Michishita et al., 2008; Kawahara et al., 2009; Tennen et al., 2011) and in DNA repair (Mao et al., 2011) suggest that it may contribute to protecting against aging and psychiatric illness by maintaining telomere integrity or protecting against and repairing the effects of oxidative stress.

Together, the occurrence of epigenetic modifications during aging and in the context of neuropsychiatric disorders may thus provide mechanistic underpinnings for the proposed age-by-disease biological interaction hypothesis, through the dual role of longevity and other age-associated genes.

## SUMMARY AND IMPLICATIONS

**FIGURE 2:**
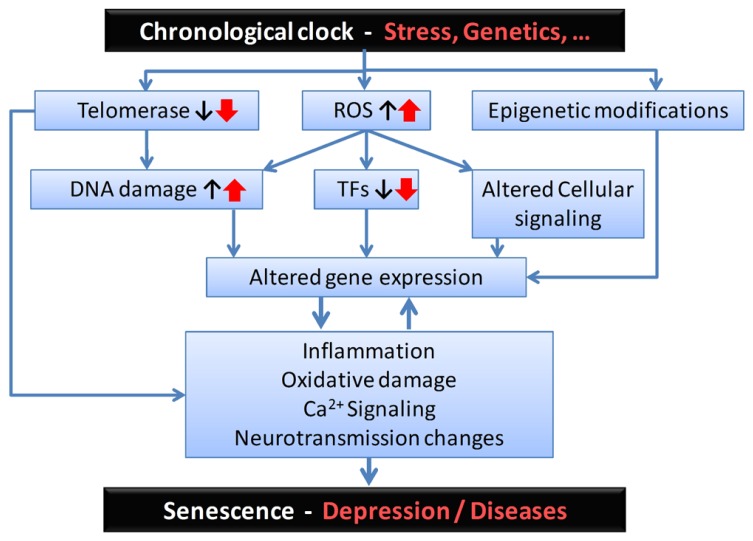
**Proposed sequence of biological events and putative mediators for the age-by-disease biological interaction hypothesis.** *From the top*: Although its biological substrates are unknown, a chronological clock drives age-related changes in gene expression. These changes can be exacerbated by psychophysiological stress and/or genetic variants, and placed on accelerated age trajectories. In this model, age-related changes in telomeres, oxidative load and epigenetic landscape, among other putative mechanisms, may represent a first level of biological events, which in turn affect basic cellular processes involved in regulating gene expression (i.e., including DNA damage, altered structure and function of transcription factors (TFs), and associated local cellular signaling). The resulting changes in the global pattern of age-dependent gene expression mediate the next set of deleterious biological events, exemplified here by increased inflammation, oxidative damage-related signaling, and changes in neurotransmission. These two levels of changes are likely to reciprocally interact. At the neural network and brain levels, the emerging properties of those specific cellular events are expressed as senescence in normal aging subjects and, in subjects at risk, as age-related symptom dimensions and diseases, including depression. Notably, the degree of individual vulnerability is thought to be under genetic and environmental control, so decreased vulnerability may mediate resiliency through the same pathways.

We propose a novel framework for investigating the development of late-life brain disorders, including LLD, which we term the age-by-disease biological interaction hypothesis. This paper expands upon an earlier version described elsewhere (McKinney and Sibille, 2012). This hypothesis posits that symptoms of LLD and other late-life brain disorders are the emerging properties of underlying biological changes, which in turn are supported by normal changes in the expression of multiple genes with age, including disease-related genes changing in disease-causing directions. Here, in addition to presenting the gene expression data on which the hypothesis is based, we discussed molecular mechanisms that may account for age-dependent gene expression changes, including loss of telomere integrity, increased oxidative stress, and epigenetic modifications. Importantly, this hypothesis complements existing hypotheses, including the vascular, inflammatory, and dementia prodrome hypotheses of LLD, but it differs in that it positions age-dependent gene expression changes as the mechanism potentially driving dysfunctions in multiple biological pathways, including vascular, inflammatory, and neurotrophic functions. A potential sequence of events is summarized in **Figure 2**. The purpose of this paper was to discuss the general framework. Examples of gene changes at the intersection of depression and aging were provided (e.g., SST and BDNF), but the exact nature and complexity of changes in multiple genes and pathways and their relevance to disease pathways will be described in details elsewhere.

The implications of this hypothesis for the prevention and treatment of LLD and other late-life brain disorders are exciting. Understanding the mechanisms mediating age-related changes in gene expression is expected to provide insight into pathophysiological mechanisms and potential targets for intervention into these disorders. Identifying key upstream hub genes mediating patterns of altered age-dependent changes would provide novel targets for further investigations. Although sirtuins and BDNF may represent obvious candidates, the large set of age-dependent genes (~10% of all genes; Erraji-Benchekroun et al., 2005) and its overlap with genes previously implicated in brain disorders (Glorioso et al., 2011) should be viewed as an enriched pool of candidate genes. Targeting such upstream factors (transcription or function) should represent productive research avenues. Early candidate interventions may include known interventions such as antidepressant medications, psychotherapy, exercise, and others. Investigating how these known interventions affect age-dependent changes in the function of critical genes may help in optimizing their implementation with respect to timing and duration of intervention for age-dependent disorders. Further, identifying genetic and environmental factors that slow or accelerate age-related changes in gene function may lead to individualized strategies aimed at promoting resilience and successful aging. Other entry points and targets for intervention are likely to arise out of understanding the mechanisms by which gene expression changes with age, including determining the role of telomere integrity, oxidative stress, and epigenetic modifications. Finally, for the broader fields of aging and gerontology, the implication of this hypothesis is that it brings together research on normal aging more closely with the investigation of neuropsychiatric and neurodegenerative diseases. Indeed, our data firmly support the assertion that they may in fact be related facets of similar biological processes, and also provide the basis for a putative mechanism of age-by-disease biological interactions.

## Conflict of Interest Statement

The authors declare that the research was conducted in the absence of any commercial or financial relationships that could be construed as a potential conflict of interest.
